# Dual radioisotopes simultaneous SPECT of ^99m^Tc-tetrofosmin and ^123^I-BMIPP using a semiconductor detector

**Published:** 2015

**Authors:** Yasuyuki Takahashi, Masao Miyagawa, Yoshiko Nishiyama, Naoto Kawaguchi, Hayato Ishimura, Teruhito Mochizuki

**Affiliations:** 1 Department of Nuclear Medicine Technology, Gunma Prefectural College of Health Sciences, Maebashi, Japan; 2 Department of Radiology, Ehime University Graduate School of Medicine, Toon, Japan; 3 Department of Radiological Technology, Ehime University Hospital, Toon, Japan

**Keywords:** Breast cancer, Myocardial perfusion, Radiotherapy, SPECT

## Abstract

**Objective(s):**

The energy resolution of a cadmium-zinc-telluride (CZT) solid-state semiconductor detector is about 5%, and is superior to the resolution of the conventional Anger type detector which is 10%. Also, the window width of the high-energy part and of the low-energy part of a photo peak window can be changed separately. In this study, we used a semiconductor detector and examined the effects of changing energy window widths for ^99m^Tc and ^123^I simultaneous SPECT.

**Methods:**

The energy “centerline” for ^99m^Tc was set at 140.5 keV and that for ^123^I at 159.0 keV. For ^99m^Tc, the “low-energy-window width” was set to values that varied from 3% to 10% of 140.5 keV and the “high-energy-window width” were independently set to values that varied from 3% to 6% of 140.5 keV. For ^123^I, the “low energy-window-width” varied from 3% to 6% of 159.0 keV and the high-energy-window width from 3% to 10% of 159 keV. In this study we imaged the cardiac phantom, using single or dual radionuclide, changing energy window width, and comparing SPECT counts as well as crosstalk ratio.

**Results:**

The contamination to the ^123^I window from ^99m^Tc (the crosstalk) was only 1% or less with cutoffs of 4% at lower part and 6% at upper part of 159KeV. On the other hand, the crosstalk from ^123^I photons into the ^99m^Tc window mostly exceeded 20%. Therefore, in order to suppress the rate of contamination to 20% or less, ^99m^Tc window cutoffs were set at 3% in upper part and 7% at lower part of 140.5 KeV. The semiconductor detector improves separation accuracy of the acquisition inherently at dual radionuclide imaging. In, this phantom study we simulated dual radionuclide simultaneous SPECT by ^99m^Tc-tetrofosmin and ^123^I-BMIPP.

**Conclusion:**

We suggest that dual radionuclide simultaneous SPECT of ^99^mTc and ^123^I using a CZT semiconductor detector is possible employing the recommended windows.

## Introduction

In nuclear cardiology, the mismatch of benzenepentadecanoicacid, 4-(iodo-123I)-b-methyl-(^123^I-BMIPP) myocardial fatty-acid metabolism single photon emission CT (SPECT) compared to technetium Tc-99m 1,2-bis (bis(2-ethoxyethyl) phosphino) ethane (^99m^Tc-tetrofosmin) myocardial perfusion gated SPECT is a good predictor of myocardial viability (1-3).

For practical reasons as well as to increase accuracy and to improve patient comfort and convenience, one-time simultaneous acquisition is desirable. But the energy resolution of an Anger camera is only about 10%, so separation of the counts from the 140.5 keV photons of 99mTc and those from the 159.0 keV photons of 123I is difficult. Up to now, the method for performing dual radioisotopes simultaneous acquisition usually relied on separating the energy windows as much as possible by narrowing one or more of the energy windows. The window width employed was 15% or 20% (4, 5), and a symmetric window was the only choice possible (6-8).

On the other hand, it is reported that the energy resolution of a semiconductor SPECT system is 5% (9). And an asymmetric window setting is a possible choice. This study investigates dual radionuclide simultaneous SPECT employing a semiconductor detector and various asymmetric window choices.

## Methods

The SPECT system used was Discovery NM 530c (GE Healthcare, Milwaukee, WI, USA) equipped with 19 pinhole collimators (9), employed list-mode raw data acquisition over 5 minutes. The matrix size was 70 × 70, and the image reconstruction voxel size was 4.0 × 4.0 × 4.0 mm. The data processor was the Xeleris (GE Healthcare, Milwaukee, WI, USA).

In this study, reconstruction was based on an implementation of a 3-D iterative Bayesian reconstruction algorithm. A Butterworth filter (order 7, cutoff frequency = 0.37 cycles/cm) was used as a post-filter (10).

### Crosstalk measurement

For crosstalk measurement using the cardiac phantom without defect, initially the crosstalk into various-sized windows was determined for both ^99m^Tc and ^123^I. The energy “centerline” for ^99m^Tc was set at 140.5 keV and that for ^123^I at 159.0 keV. For ^99m^Tc, the part of the window from the “centerline” down to a low-energy cutoff (the low-energy-window width) was set to values that varied from 3%-10% of the ^99m^Tc photopeak energy and the part of the window from the centerline up to a high-energy cutoff (the high-energy-window width) was independently set to values that varied from 3%-6% of the ^99m^Tc photopeak energy. On the other hand, the window-width variations for ^123^I covered a larger range on the high energy side and a smaller range on the low energy side: the low energy-window-width varied from 3%-6% of 159.0 keV and the high-energy-window width from 3% to 10% of 159.0 keV.

After reduction of the counts of ^123^I within energy window of the ^99m^Tc, the presence of down-scattered ^123^I counts subtracted by the dual energy window (DEW) method (11). The energy window width for scatter correction is 120 keV±5%.

**Figure 1:**
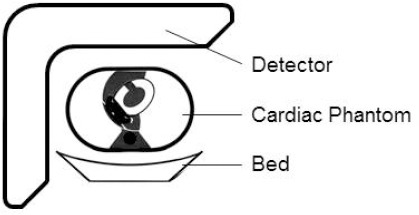
Geometric arrangement of the c cardiac phantom study

In one initial study, we used the cardiac phantom (HL type, Kyoto-kagaku, Kyoto, Japan). The rate of crosstalk and the concentration linearity were analyzed with the data obtained from the cardiac phantom. The myocardium was set to the center of effective field of view. The acquisition position is shown in Figure 1.

**Figure 2:**
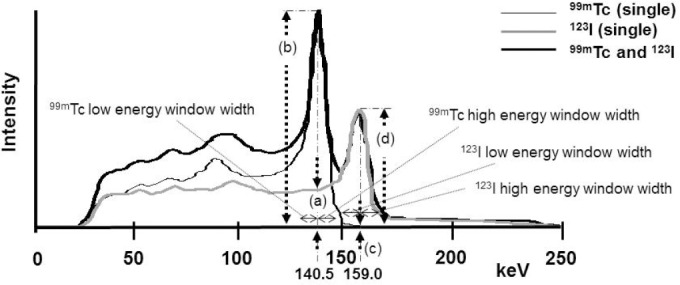
The energy spectrum of ^99m^Tc and ^123^I by the Discoverly NM530c. Intensity was made similar to the clinical study

The radionuclide injected to the phantom was based on previous human studies that injected 1.8% (12) of 259 MBq of ^99m^Tc-Tetrofosmin and 5.4% (13) of 111 MBq of ^123^I-BMIPP was accumulated in the myocardium. Therefore, the injection rate was set to 45.0 kBq/ml, nearly same. Single nuclide and dual simultaneous energy spectrum is shown in Figure 2.

The measurement itself involved using either the cardiac phantom of ^99m^Tc, or the cardiac phantom of ^123^I. The count rate results were appropriately normalized for the activity levels. After that normalization, it was possible to compute the ratio of the count rate of ^123^I photons in the window centered on the ^99m^Tc photopeak, defined as ^123^I (140KeV), divided by the count rate of ^99m^Tc photons in the window centered on its own photopeak, defined as ^99m^Tc (140KeV). The ratio then can be represented by ^123^I (140KeV) / ^99m^Tc (140KeV); (equal to a/b in Figure 2). This ratio was found with different settings of the ^99m^Tc windows (low energy part and high energy part). This was the iodine to technetium-window crosstalk. It was also possible to compute a similar crosstalk ratio in the opposite direction, the technetium in the iodine window crosstalk. The ratio then can be represented by ^99m^Tc (159KeV) / ^123^I (159KeV); (equal to c/d in Figure 2) count ratio. The count per pixel is the average.

### The linearity of the concentration

Another experiment was performed to check the linearity of image results when the concentration of each radionuclide was varied. The cardiac phantom used contained only a single radionuclide or a mixture of both (dual) radionuclides. The concentration of the cardiac phantom with only ^99m^Tc or only ^123^I was 20, 40, 60, 80 or 100% of 45.0 kBq/ml. A mixture of both (dual) radionuclides, the concentration of ^99m^Tc + ^123^I were 20%+80%, 40%+60%, 60%+40% and 80%+20%, respectively.

### Selection of the energy window width

The following points were considered in coming to a recommendation for the window settings for ^99m^Tc, (1) The iodine to technetium crosstalk should be 20% or less, (2) not too many potential true counts should be lost, (3) we do not want the high-energy cutoff for the technetium window to overlap the low-energy cutoff of the iodine window. On the other hand, for ^123^I the following points were considered; (1) Although the ratio for technetium-to-iodine-window crosstalk was almost constant; not too many potential true counts should be lost, (2) stability of counts was observed for a high-energy-window width greater that 7%; (3) We do not want the low-energy cutoff for the iodine window to overlap the high-energy cutoff of the technetium window.

### The cardiac phantom study

**Figure 3:**
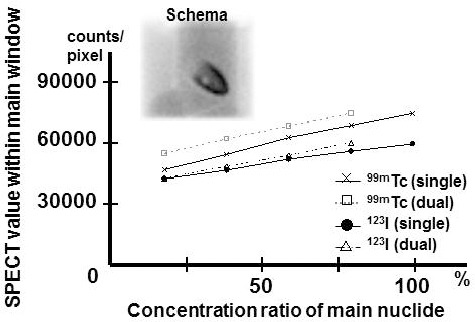
Schema is the image of the cardiac phantom with injection radionuclide. This static image was acquired by an Anger type gamma camera. The count ratio of single radionuclide and a mixture of both radionuclides to which concentration was changed. X-axis: Concentration of ^99m^Tc or ^123^I in a single radionuclide (100%) and a mixture of both radionuclides (20, 40, 60 and 80%). Y-axis: The count of the point source per pixel

The cardiac phantom study placed 1.5 cm, 3.0 cm and left anterior descending defect into the anterior, and compared the detectability of that defect under various conditions. Only ^99m^Tc (single), only ^123^I (single), or a mixture of both (dual) radionuclides was injected into the phantom. ^99m^Tc and ^123^I of 45.0 kBq/ml, the same volumes were injected into the myocardium. And the same volumes of 10.0 kBq/ml (14) were injected into the lung, the LV cavity, the mediastinum and the liver (Figure 3.upper left). This static image (schema) was acquired by an Anger type gamma camera (Infinia; GE Healthcare, Milwaukee, WI, USA).

The count for the anterior view within the ^99m^Tc window averaged 600 counts/pixel and that within the ^123^I window averaged 400 counts/pixel. The acquisition count was similar to the clinical study. Single radionuclide was decided that the high-energy-window width should be 5% and the low-energy-window width should be 5%. This condition is conventional symmetrical window width.

### Human study

A 54-year-old man with hypertrophic cardiomyopathy participated in this study. Informed consent was obtained after a detailed explanation of the purpose of the study and scanning procedures. This patient was injected with 111 MBq of ^123^I-BMIPP at rest and SPECT imaging was performed 20 minutes after injection. After completing ^123^I-BMIPP SPECT, 295 MBq of ^99m^Tc-tetrofosmin was administered to obtain simultaneous ^123^I-BMIPP and ^99m^Tc-tetrofosmin SPECT. As in the myocardial phantom studies, the protocols given above were employed.

## Results

### Energy spectrum

The energy spectra for single radionuclide acquisitions and for a dual radionuclide simultaneous acquisition are shown in Figure 2. Although it is the same activity, energetic differs.

### Result of crosstalk measurements

**Figure 4:**
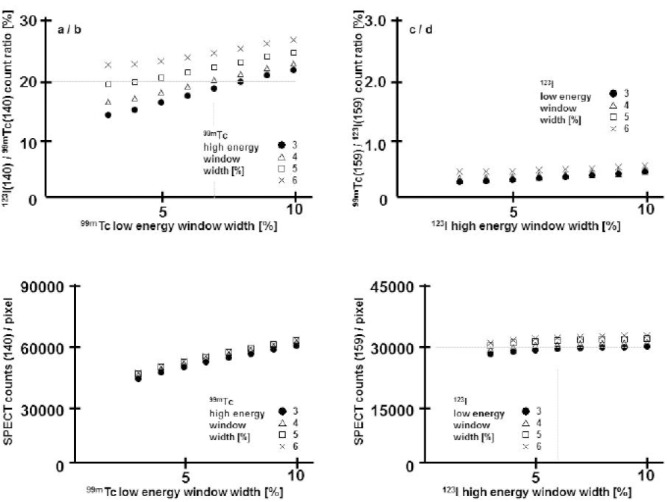
In crosstalk measurement using cardiac phantom, the crosstalk into various-sized windows was determined for both 99mTc and 123I.

The technetium in the iodine window crosstalk leads to a ratio for ^99m^Tc (159) divided by ^123^I (159) that is 1% or smaller (Figure 4.upper right). However, the iodine in the technetium window crosstalk leads to a ratio for ^123^I (140) divided by ^99m^Tc (140) that is above 20% for most of the choices for the technetium windows (Figure 4. upper left).

Based on these initial results, it was decided that the high-energy-window width should be 3% and the low-energy-window width should be 7% for ^99m^Tc. Also it was decided that the high-energy-window width should be 6% and the low-energy-window width should be 4% for ^123^I.

a. The rate in which the counts of 123I crosstalk to the window of 99mTc(140)
b. The rate in which the counts of 99mTc crosstalk to the window of 123I(159)
c. The count of the 99mTc(140) window including the count of 123I(140) crosstalk to which the 99mTc window width was changed
d. The count of the 123I(159) window including the count of 99mTc(159) crosstalk to which the 123Tc window width was changed

In concentration change study, the count of each single radionuclide was compared with the count of a mixture of both radionuclides. The count was measured every 20% and it had good linearity (Figure 3). The increase in crosstalk was remarkable by an increase in ^123^I (140) / ^99m^Tc (140) concentration.

### The cardiac phantom study

Bull’s eye map of a cardiac phantom without defect was compared with three pattern of defects of the cardiac phantom regarding distribution of the tracer.

**Figure 5:**
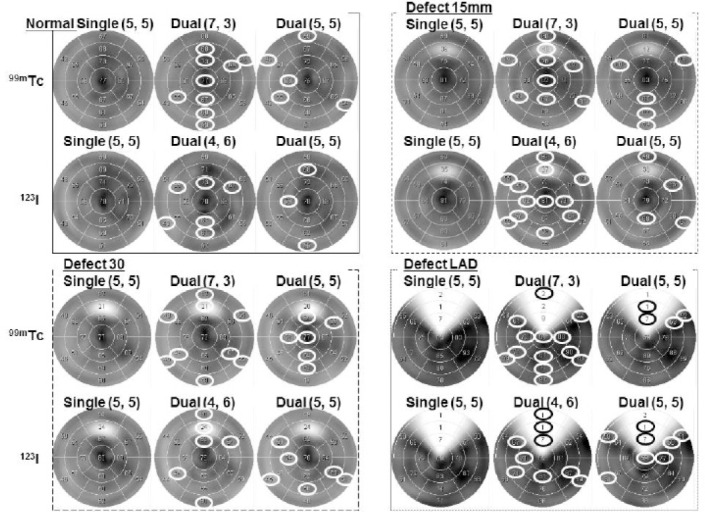
Bull’s eye map for the cardiac phantom with anterior defect in single- and dual-radionuclide. The defect is 1.5 cm (upper right), 3.0 cm (lower left) and left anterior descending (lower right). Upper left image was without defect

For distribution of the tracer we used the contrast ratio divided 17 segments. Bull’s eye map were produced for single ^99m^Tc in the phantom, a ^99m^Tc image from a mixed radionuclide phantom (dual ^99m^Tc image), a ^123^I image from a mixed radionuclide phantom (dual ^123^I image), and an image with single ^123^I in the phantom (Figure 5). Bull’s eye map of single ^99m^Tc was similar to dual ^99m^Tc (7-3). Also Bull’s eye map for single ^123^I was similar to dual ^123^I (4-6). Without defect and with defect size of 1.5 cm had similar ^99m^Tc single image (white and black line oval).

### Human study

The energy window width that image reconfiguration used was: ^99m^Tc photo peak 140.5 keV, high-energy-window width 3%, low-energy-window width 7%, and ^123^I photo peak 159.0keV, high-energy-window width 6% and low-energy- window width 4%.

### An example of a mismatch between perfusion and ^123^I-BMIPP

**Figure 6:**
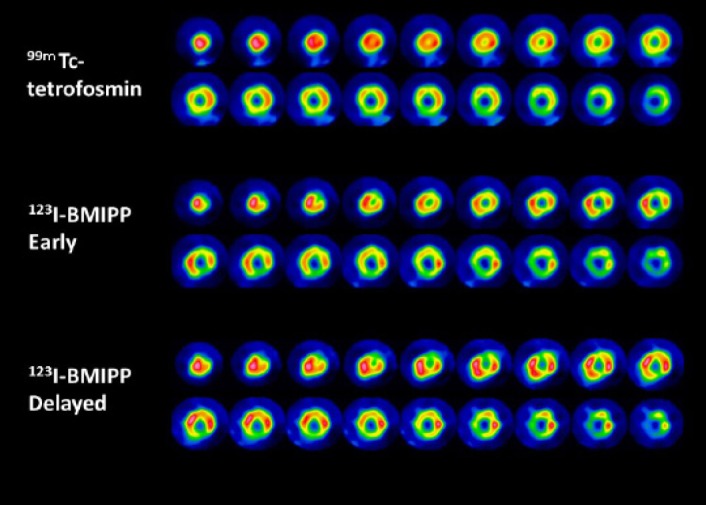
^99m^Tc-tetrofosmin and ^123^I-BMIPP are displayed (top: ^99m^Tc-tetrofosmin, middle: ^123^I-BMIPP in early phase, and bottom: ^123^I-BMIPP in delayed phase)

Images from our 54-year-old patient with hypertrophic cardiomyopathy using dual isotope SPECT images with ^99m^Tc-tetrofosmin and ^123^I-BMIPP are displayed in Figure 6 in short-axis views. ^123^I-BMIPP uptake was moderately to severely reduced from anterolateral to apical and inferior region, while ^99m^Tc-tetrofosmin uptake is slightly decreased or almost normal in basal anterior area and apex. Invasive coronary angiography was normal (not shown).

## Discussion

Dual radionuclide simultaneous acquisition of ^99m^Tc and ^123^I uses the technique of separating an energy window as much as possible. Usually energy window width must be symmetrical in the high- energy-window and low- energy-window parts. When energy window width is 15% or 20% (4, 5), energy window width overlaps.

Therefore, acquisition energy peak which ^99m^Tc should move to low energy side, and ^123^I should move to high energy side, to prevent overlap. However using that technique, acquisition count decreases remarkably and quality of image deteriorated. As energy resolution was not optimal, perfect separation was difficult.

As for the energy window width of Discovery NM530c system, it can be changed symmetrically and freely. It is not necessary to shift a photo peak and an energy window can be separated. Therefore, it is possible to acquire as many photons as possible in an efficient manner. Since sensitivity is better with a semiconductor detector than with an Anger camera, one can acquire sufficient counts even if an energy window was narrow.

We calculated in consideration of energy resolution and the rate of crosstalk according to the phantom study the suitable window width. ^99m^Tc energy window were photo peak 140.5 keV, high-energy-window width 3%, low-energy-window width 7%, and ^123^I energy window were photo peak 159.0 keV, high-energy-window width 6% and low-energy- window width 4%. The change of energy window width (4, 5) does not largely influence the single nuclide image.

Additionally in the linearity study of the concentration, a mixture of both radionuclides of the same concentration increased the count rate about 20% compared with the count of ^99m^Tc only single radionuclide. The Compton scattering of ^123^I is included in the crosstalk to a main energy window of the ^99m^Tc, about this, it considers the DEW subtraction method (11).

Uptake rates differ remarkably by the radionuclide. The uptake rate of ^99m^Tc-tetrofosmin is 1.8% (12) and ^123^I-BMIPP is about 5.1% (13). The dose (accumulation) to ^123^I-BMIPP is nearly equal to ^99m^Tc-tetrofosmin. However, the energy spectrum of the energetics of ^123^I is only 50% or less by the intensity of ^99m^Tc. For this reason, we have to make window width change according to the activity. It can be imagined that it is satisfactory even if it changes a small percent for the window width for this result.

In this study we showed that dual radionuclide separated well according to the presented technique. We started to use this technique for many clinical studies.

## Conclusion

A semiconductor detector is better in energy resolution and sensitivity compared to the conventional Anger type detector. Therefore, energy window width could be narrowed and it was possible for dual radionuclide simultaneous SPECT by ^99m^Tc and ^123^I.

## References

[1] Dobbeleir AA, Hambys ASE, Franken PR (1999). Influence of methodology on the presence and extent of mismatching between ^99m^Tc-MIBI and ^123^I-BMIPP in myocardial viability studies. J Nucl Med.

[2] Tamaki N, Tadamura E, Kawamoto M Magata, Yonekura Y, Fujibayashi Y (1995). Decreased uptake of iodinated branched fatty acid analog indicates metabolic alterations in ischemic myocardium. J Nucl Med.

[3] Kumita S, Mizumura S, Kijima T, Machida M, Kumazaki T, Tetsuou Y (1995). ECG-gated dual isotope myocardial SPECT with ^99m^Tc-MIBI and ^123^I-BMIPP in patiens with ischemic heart disease. Kaku Igaku.

[4] (1994). National Electric Manufacturers Association (NEMA). Performance Measurements of Scintillation Cameras. Standards publication NU-1–1994.

[5] (2001). NEMA Standard Publication NU 1-001, Performance Measurements of Scintillation Cameras.

[6] Mizumura S, Kumita S, Kumazaki T (1995). A study of the simultaneous acquisition of dual energy SPECT with ^99m^Tc and ^123^I: Evaluation of optimal window setting with myocardial phantom. Kaku Igaku.

[7] Hirata M, Monzen H, Suzuki T, Ogasawara M, Nakanishi A, Sumi N (2009). Evaluation of a new protocol for two-isotope ^123^I-BMIPP/^99m^Tc-TF single photon emission computed tomography (SPECT) to detect myocardial damage within one hour. Jpn J Med Phys.

[8] Inoue T (1993). Basic study of dual radionuclide data acquisition with Tc-99m and I-123 to establish quantitative brain SPECT. Ehime Medical Journal.

[9] Bocher M, Blevis IM, Tsukerman L, Shrem Y, Kovalski G, Volokh L (2010). A fast cardiac gamma camera with dynamic SPECT capabilities: design, system validation and future potential. Eur J Nucl Med.

[10] Takahashi Y, Miyagawa M, Nishiyama Y, Ishimura H, Mochizuki T (2013). Performance of a semiconductor SPECT system: comparison with a conventional Anger-type SPECT instrument. Ann Nucl Med.

[11] Jaszcak RJ, Greer KL (1984). Improved SPECT quantification using compensation for scattered photons. J Nucl Med.

[12] Kubo A, Nakamura K, Hashimoto J, Sammiya T, Iwanaga S, Hashimoto S (1992). Phase I clinical trial of a new myocardial imaging agent, 99mTc-PPN1011. Kaku Igaku.

[13] Torizuka K, Yonekura Y, Nishimura T, Tamaki N, Uehara T, Ikekubo K (1991). A Phase 1 study of beta-methyl-p-(123I)-iodophenyl-pentadecanoic acid (123I-BMIPP). Kaku Igaku.

[14] Hatakeyama R (2001). Heart phantom with liver object. A new textbook of nuclear medicine technology.

